# The Effects of Thermophysical Properties and Environmental Conditions on Fire Performance of Intumescent Coatings on Glass Fibre-Reinforced Epoxy Composites

**DOI:** 10.3390/ma8085216

**Published:** 2015-08-11

**Authors:** Baljinder K. Kandola, Piyanuch Luangtriratana, Sophie Duquesne, Serge Bourbigot

**Affiliations:** 1 Institute for Materials Research and Innovation, University of Bolton, Bolton BL3 5AB, UK; E-Mail: piyanulu@scg.co.th; 2 Unité Matériaux et Transformations (UMET)-CNRS UMR 8207-Group Reaction and Resistance to Fire (R2Fire), École Nationale Supérieure de Chimie de Lille, University of Lille, Avenue Mendeleiev, CS 90108, 59652 Villeneuve d'Ascq Cedex, France; E-Mails: sophie.duquesne@ensc-lille.fr (S.D.); serge.bourbigot@ensc-lille.fr (S.B.)

**Keywords:** intumescent coatings, char expansion, cone calorimetry, thermal conductivity at high temperatures, durability, mechanical properties

## Abstract

Intumescent coatings are commonly used as passive fire protection systems for steel structures. The purpose of this work is to explore whether these can also be used effectively on glass fibre-reinforced epoxy (GRE) composites, considering the flammability of the composites compared to non-flammable steel substrate. The thermal barrier and reaction-to-fire properties of three commercial intumescent coatings on GRE composites have been studied using a cone calorimeter. Their thermophysical properties in terms of heating rate and/or temperature dependent char expansion ratios and thermal conductivities have been measured and correlated. It has been suggested that these two parameters can be used to design coatings to protect composite laminates of defined thicknesses for specified periods of time. The durability of the coatings to water absorption, peeling, impact, and flexural loading were also studied. A strong adhesion between all types of coatings and the substrate was observed. Water soaking had a little effect on the fire performance of epoxy based coatings. All types of 1 mm thick coatings on GRE helped in retaining ~90% of the flexural property after 2 min exposure to 50 kW/m^2^ heat flux whereas the uncoated laminate underwent severe delamination and loss in structural integrity after 1 min.

## 1. Introduction

Fiber-reinforced polymeric composites for structural applications are required to conform to specific fire performance requirements and to retain their mechanical integrity after exposure to heat/fire. Unlike steel and other metals, many polymeric composites will lose their structural integrity when exposed to temperatures close to the glass transition temperature of the resin matrix. The most effective technique of protecting these materials against heat and fire is the use of surface coatings. Intumescent coatings are designed to expand to form an insulating and fire resistant charred barrier between the fire and the underlying substrate. This char protects the underlying structure, acting as a thermal insulator/barrier against flame and heat by physically stopping the heat and oxygen to penetrate the materials. The thickness, coherence and porosity of the char determine its thermal barrier efficiency [1,2]. These coatings are available as solvent-based or water-based systems, which can be applied in an economical and simple way, such as by spray, brush or roller, onto several materials including metallic materials, polymers, textiles and wood. A typical intumescent system contains three active components [3] bound together by a binder. The active components include: (i) A carbonization agent (carbonific), which is carbon rich polyhydric compound that influences the amount of char formed and the rate of char formation. (ii) An acid source, usually containing or generating poly (phosphoric acid) or other acid which promotes char formation and (iii) a blowing agent, which decomposes and releases non-flammable gases (e.g., CO_2_, H_2_O and NH_3_) that expands the char and forms a swollen multi-cellular layer, such as melamine, which on heating releases NH_3_. A chemical interaction between these components leads to an intumescent char [4,5]. The rate and degree of char expansion depends upon many factors such as heating conditions, type of binder, solvent used, and any other additive or primer used for binding the coating to the substrate.

Although intumescent coatings can provide good thermal barrier performance, there are some disadvantages. For instance, the high loadings required to achieve the necessary level of performance leading to viscous paints and hence, altering the properties of the substrate. Also, the water soluble nature of water based intumescent coating types will permit leaching, affecting the durability of those coatings.

Traditionally intumescent coatings are used as “passive fire protection” systems for structural materials such as steel [6] and are expected to maintain their structural integrity between 1 and 3 h when the temperature of the surroundings is in excess of 1100 °C. Resistance to fire of intumescent coatings on steel structures is typically measured by temperature *versus* time curves from large scale such as UL 1709 [7], BS EN 13381-8:2010 [8] and ISO 834-10:2014 [9]. Test results for commercial coatings are easily available from literature [10] or from intumescent coating manufactures. The large scale tests however, are expensive, require large amounts of materials, and provide limited scientific knowledge required for product development. Hence, many researchers have attempted to mimic these large tests on bench scale using either a burner [5] or a furnace [11] or even a cone calorimeter at heat fluxes varying from 25–75 kW/m^2^ [12,13,14]. The limitation of the cone calorimeter however, is that the surface temperature does not exceed 750 °C [13], hence results cannot be correlated with large scale tests.

These coatings and test methods are designed for steel structures, which are non-flammable. For flammable substrates these coatings have to be more efficient to further protect the substrate from decomposition and/or ignition. Here we study the reaction to fire properties of three commercial coatings on glass fiber-reinforced composites using a cone calorimeter and their thermal barrier properties by using thermocouples on the coating-composite interface and on the back surface of composites. We also have quantified their char expansion under different heating conditions using methodology discussed in detail elsewhere [2], measured their thermal conductivities at room and elevated temperatures (methodology developed and discussed in [2]), and established relationship between thermal conductivity and char thickness to set a benchmark for the requirements of on ideal coating for a composite laminate of a defined thickness. The three intumescent coatings are identified in the text based on the binder used in each type. Two were epoxy resin binder based, identified as epoxy based intumescent coating (EI) and flame retarded epoxy based coating (EDI), in the latter the epoxy resin is modified with DOPO (9,10-Dihydro-9-oxa-10-phosphaphenanthrene-10-oxide). The third coating containing vinyl acetate/vinyl ester copolymer system is water based, identified here as water based intumescent coating (WI). The effects of different environmental conditions on durability and fire performance of these coatings have also been studied in order to evaluate their practical and commercial viability.

## 2. Results and Discussion

### 2.1. Thermal Barrier Performance of Coatings

**Figure 1:**
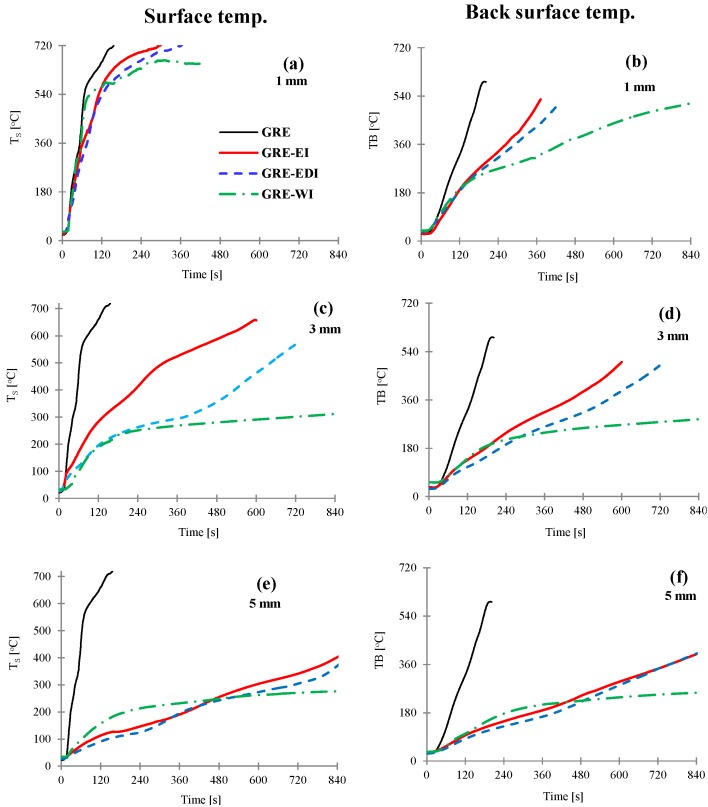
The surface (**a**,**c**,**e**) and back surface temperatures (**b**,**d**,**f**) *vs.* time for control and GRE-EI, GRE-EDI and GRE-WI coated samples at 50 kW/m^2^. Coating thickness = 1 mm (**a**,**b**), 3 mm (**c**,**d**) and 5 mm (**e**,**f**). GRE: glass fibre-reinforced epoxy; EI: epoxy based intumescent coating; EDI: flame retarded epoxy based intumescent coating; WI: water based intumescent coating.

As mentioned above, the thermal barrier efficiency of intumescent coatings on steel structures is usually evaluated from temperature-time curves obtained from thermocouples embedded on the unexposed surface of steel plate in fire tests and determination of the “time to failure” in terms of time to reach a given temperature, defined by a specific test [7,8]. In UL 1709 test, for example the coated surface is exposed to 147 kW propane jet burner that produces 180 kW/m^2^ heat flux at the exposed surface and the coating is expected to hold the back surface temperature of the substrate to a maximum of 180 °C for 30 min. This criterion in the test is used because the structural steel loses its load bearing capacity when the temperature exceeds 500 °C. However, in case of composites when the temperature reaches the softening or the glass transition temperature (*T*_g_) of the resin, ~50% of mechanical properties of the laminate are lost [15], increasing progressively with rise in temperature leading to 100% loss by decomposition/burning of resin. As an effective thermal barrier protection, the coating should stop or retard the temperature rise at the surface to reach *T*_g_ or the pyrolysis temperature (the temperature at which decomposition of the resin starts) of the epoxy resin. The *T*_g_ of epoxy resin is 150–220 °C, for the resin used in this work is 180 °C, while the onset of decomposition temperature is 250 °C. In this work we have used a cone calorimeter to determine thermal barrier efficiency of these coatings, keeping external heat flux 50 kW/m^2^ (commonly used heat flux to test reaction-to-fire properties of composites in developing fires) and measuring the temperature profiles by inserting thermocouples under the coatings and on the back surfaces of the laminates. In cone calorimetric testing of intumescent coatings, usually the distance between the cone heater and the sample is increased from standard 25 to 50 mm [13] or other suitable distance to compensate for the expansion of the coating [14]. The distance was kept to standard 25 mm in this case as our primary interest was to keep the heat flux constant and study the rate of temperature rise of composite surface during early stages of the char expansion and also to measure the time taken for top and back surfaces of the laminate to reach 180 or 250 °C. The temperatures profiles for 1, 3 and 5 mm thick coatings were noted and are shown in Figure 1. The thermocouple was placed on the surface, hence is an estimated surface temperature, whereas, for coated laminates, the temperature measured was underneath the coating and represents the temperature at the interface of the laminate and the coating. Times to reach each surface 180 or 250 °C are reported in Table 1.

As can be seen from Figure 1a that the surface temperature of the control GRE composite rises sharply reaching softening temperature within 28 s (Table 1) and the highest value of 720 °C only after 156 s, which indicates very high rate of temperature rise at the surface of uncoated GRE composite. The back surface temperature rise is slower than the surface temperature as can be seen from the slope of the curve, reaching softening temperature within 76 s and maximum 590 °C after 191 s. With 1 mm thick coating the temperature rise on the surface (Figure 1a) is similar to the control for ~30 s, and once the coating is expanded some difference in the temperature can be seen. The temperature rise of the back surface is much slower. All three coatings behave similarly for first 156 s (Figure 1b), then the thermal barrier efficiency depends upon the expanded char. The effect is more pronounced as the coating thickness and the char thickness increases. The efficiency of coatings in terms to time to reach 180 or 250 °C is as: WI > EDI > EI.

This effect is directly proportional to the thickness of the char as seen in Table 1. As the thickness gets to 5 mm, all charred structures are thick enough to act as efficient thermal barriers as can be seen from Figure 1e,f.

**Table 1:** Thermal barrier properties of different intumescent coatings of varying thicknesses on glass fibre-reinforced epoxy (GRE) composite samples, exposed to 50 kW/m^2^ heat flux.

Sample	Coating Thickness (mm)	Char Thickness		Time to Reach 180 °C (s)		Time to Reach 250 °C (s)	
		Exposure time (s)	Char thickness (mm)				
				Surface	Back	Surface	Back
Control	-	200	-	28	76	37	95
GRE-EI	1.27 ± 0.01	500	6.8 ± 2.2	33	114	43	164
	2.96 ± 0.16	900	10.0 ± 2.0	65	176	98	258
	5.28 ± 0.22	1400	15.8 ± 0.9	338	472	471	495
GRE-EDI	1.35 ± 0.02	500	9.8 ± 2.8	38	107	56	152
	2.95 ± 0.09	900	20.7 ± 1.3	107	222	208	339
	5.72 ± 0.09	1600	27.2 ± 2.7	337	507	507	537
GRE-WI	0.94 ± 0.03	800	24.1 ± 2.6	32	109	48	193
	2.91 ± 0.09	800	41.7 ± 2.4	110	166	236	438
	5.25 ± 0.82	1200	36.3 ± 4.5	153	494	494	759

As seen from Table 1 when the samples is protected by 1 mm thick coating, there is no significant delay in time (~5–10 s on surface and 31–38 s back surface) to reach 180 °C, however, a 3 mm thick coating significantly delayed the time to reach 180, with further delay in 5 mm coatings. A similar effect is seen for the time required to reach the pyrolysis temperature of the resin (250 °C) on the surface and back surface for all coated samples. That means with the 1 mm coatings of GRE-EI, GRE-EDI and GRE-WI, the GRE composite will start losing its mechanical properties in a short period of time after exposure to a high heat source. Coatings of ≥3 mm thickness could provide longer time to retain the mechanical properties. However, on a 3 mm thick laminate it is impractical to have a coating of >1mm thickness, hence the rest of the work was done on 1 mm thick coatings.

### 2.2. Thermo-Physical Properties of Intumescent Char

The basic principle of an intumescent coating is that on exposure to heat it expands and the low thermal conductivity of the expanded char provides a thermal barrier effect. Hence, quantitative information about both of these parameters can help in designing and developing new coatings.

Due to commercial sensitivity of the coatings used here, their exact compositions are not provided. Hence, results cannot be analyzed in terms of their compositions and mechanism of intumescence. In general the expansion of an intumescent coating after activation by fire or heat occurs as a sequential chemical reaction between the three active components (acid source, carbonific, and blowing agent), which generally involves the decomposition of the acid source to generate a mineral acid, and then the acid reacts with the carbonization agent to form the carbonaceous char, while the blowing agent generates the gases. The formed gases expand the char. The swelling occurs due to the gases released following heating becoming trapped in the viscous fluid char layer and increasing the volume of the coating, and then a multi-cellular char structure of low thermal conductivity containing voids (bubbles) is formed. The expansion continues until the blowing agent is exhausted, or the solid matrix is insufficiently elastic. The polymer binder used in a coating on heating promotes crosslinking reactions, and hardens the structure. Type of polymer binder used also affects the char expansion, its porosity and hence, thermal conductivity as is the case in EI and EDI coatings, which are similar in composition and the binder (epoxy resin) type except that in EDI epoxy resin is reacted with DOPO.

**Figure 2:**
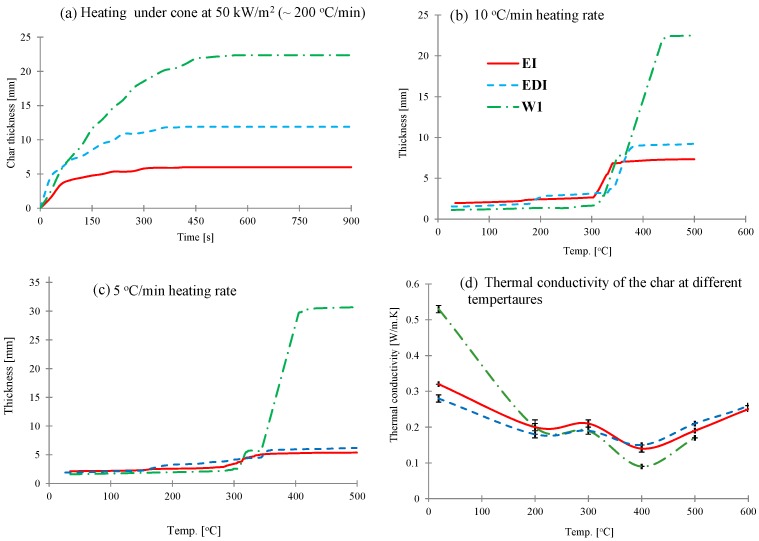
(**a**–**c**) The intumescent coatings’ expansion: (**a**) On the substrate as a function of time when exposed to a radiant heat of 50 kW/m^2^ in a mass loss calorimeter. (**b**,**c**) As a function of temperature at 10 and 5 °C/min respectively in a rheometer and (**d**) thermal conductivities as a function of temperature obtained by hot disk method.

The char expansion of these intumescent coatings under different conditions was measured. The results for expansion as a function of time measured during mass loss calorimeter experiments at 50 kW/m^2^ using an infrared camera (discussed in details elsewhere [2]) are shown in Figure 2a. In this experiment, a constant heat flux is applied to the surface of the sample and with ~200 °C/min heating rate (estimated from the slopes of temperature profiles in Figure 1) the char expands very quickly and once the surface temperature stabilizes, the char expansion/oxidation behavior becomes time dependent. As can be seen from Figure 2a, in this experiment both EI and EDI coatings started expanding immediately and rate of expansion was very high until 80 s, after which expansion was slower and became constant after 300 s. For WI coating, the char kept expanding until 450 s and then stopped.

However, when these coatings (without a substrate) are heated in a rheometer, where the heating is uniform on all sides and the heating rate is very low (5 and 10 °C/min), the expansion behaviors of three coatings are very different. All three intumescent coatings expand in two distinct stages; in the first stage char expansion is slow, whereas in the second stage expansion rate is much higher (Figure 2b,c). In epoxy based EI and EDI coatings, the first significant expansion started at ~180 °C and then the second expansion occurred at ~300 °C. On the other hand, water based WI water coating started expanding at ~300 °C, followed by the second major expansion occurring after 350 °C. The effect of heating rate on char expansion of these intumescent coatings can also be observed from Figure 2b,c. At 10 °C/min heating rate the expansion for all three coatings follows a similar pattern as seen from cone calorimetric results, although the actual values and rate of expansion are different due to different heating regimes. However, on reducing heating rate to 5 °C/min, the trend is very different, *i.e.*, while the expansion of EI and EDI slightly decreased from those at 10 °C/min, it increased in WI coating. This is clearer from Table 2, where the char expansion ratios (char thickness/coating thickness) are presented. This different trend is probably due to the EI and EDI being epoxy based intumescent coating systems, require a higher heat treatment to build up the char, whereas the water based intumescent coating (WI) could char better at the lower heating condition.

As seen from Table 2, while the expansion ratios are different depending on the heating conditions, the trend for expansion is similar in all cases as: WI > EDI > EI, which indicates that WI coating can provide higher expanded char compared to EI and EDI coatings under all conditions. The thermal barrier efficiencies of these three coatings however, are very similar for first ~150 s as seen from Figure 1b, also indicated by the time taken to reach surface temperature of the laminate 180 (glass transition temp of resin) or 250 °C (decomposition temperature of the resin) from Table 2, which is due to the fact that it is not just the thickness but also the thermal conductivity, which affects the heat transfer through the coating.

**Table 2:** The char expansion ratios from different experiments using different heating conditions.

Testing	Char Expansion Ratio *		
	EI	EDI	WI
Cone calorimetry	5.3	7.2	25.6
Mass calorimeter	5.8	7.2	35.2
Rheometer: 10 °C/min	4.0	5.1	20.7
Rheometer: 5 °C/min	2.6	3.1	22.1

* $\text{Char expansion ratio}=\frac{\text{Char thickness}}{\text{Coating thickness}}$.

The thermal conductivity values of the three coatings at different temperatures varying from room temperature to 700 °C by using hot disk method (measured at 100 °C intervals) are plotted in Figure 2d. The thermal conductivity values for all intumescent chars vary from 0.1 to 0.4 W/mK, which is similar to that as reported in literature [16,17]. While at room temperature, WI coating has the highest thermal conductivity value of 0.53 W/mK than EDI (0.32 W/mK) and EI (0.27 W/mK), the values at higher temperatures differ less from each other. The thermal conductivities of these three coatings first decreased and then increased with increasing temperature (Figure 2d). The first significant drop was observed at 200 °C, which remained constant until 300 °C and then decreased again, the minimum being at 400 °C, after which the values increased and kept increasing until 700 °C. This behavior is characteristic of an intumescent material, when exposed to high temperature the material expands and forms a porous char structure of low heat conductivity. Hence, as the char expands due to increase in porosity the thermal conductivity decreases. Above 400 °C, the char structure does not change significantly and hence thermal conductivity increases as a function of temperature [16]. This behavior corroborates very well with the char expansion study shown in Figure 2b. However, despite different char thicknesses, their thermal conductivities are very similar, which is due to the fact that while thermal conductivity is the material’s intrinsic property, it also depends upon the char structure and its porosity. These parameters are useful in designing surface coatings that can protect a particular substrate from a defined thermal threat for a specified period of time. For example, in case of composites used in this work, since glass transition temperature and decomposition temperature of the epoxy resin are ~180 and 250 °C, a temperature point at 200 °C was selected to evaluate the minimum requirement for both of thermal conductivity and coating thickness in order to protect the underlying composite structure. The results from Figure 2 and Table 3 indicate that ~0.2 W/mK is the minimum thermal conductivity value of the char that should be able to protect a composite structure from heat to maintain structural integrity for a period of time.

**Table 3:** The thermal conductivity and char thickness requirements of intumescent coatings to protect the composite surface temperature to reach 200 °C.

Coating	Surface Temperature of the Laminate, underneath the Coating (°C)	Char Thickness (mm)	Thermal Conductivity (W/mK)
EI	200	3.2	0.20
EDI	200	5.1	0.18
WI	200	6.5	0.20

### 2.3. Durability of Coatings

#### 2.3.1. Durability to Peeling: Adhesion between Coatings and GRE Surfaces

In Table 4 mass loss (%) and coating peeled (%) off after the tape pull test for all coated samples are reported. The results show that none of the coating peeled off, indicating a good adhesion strength at the interface between coating and substrate. This is expected as the epoxy resin used as binder in EI and EDI coatings and acrylic resin in WI coatings are compatible with the epoxy resin used in the substrate (GRE).

**Table 4:** The mass loss (%) of the intumescent coating after the tape pull and water-soak tests.

Sample	Tape Pull Test		Water Soak Test for 24 h			
	Mass Loss (%)	Peeling (%)	Drying at RT 24 h		Drying at 100 °C for 2 h	
			Mass Loss (%)	Change in Thickness (%)	Mass Loss (%)	Change in Thickness (%)
GRE-EI	0.01 ± 0.01	-	0.22 ± 0.06	−0.52 ± 0.15	0.48 ± 0.07	−0.59 ± 0.25
GRE-EDI	0.01 ± 0.01	-	0.49 ± 0.28	−1.14 ± 0.24	0.76 ± 0.30	−1.34 ± 0.26
GRE-WI	0.01 ± 0.01	-	4.18 ± 0.28	−7.28 ± 0.70	4.36 ± 0.29	−7.35 ± 0.76

The (−) sign represents reduction in coating thickness. RT: room temperature.

#### 2.3.2. Durability to Water

The effect of water on the durability of these coatings was investigated using the water-soak test. In this experiment, all edges were sealed with an epoxy resin prior to the test to avoid the water absorption into the laminated structure. The changes in mass before and after the test are reported in Table 4. The results show that after the water-soak test for 24 h and drying the samples at room temperature for 24 h, GRE-EI and GRE-EDI samples coatings had minimal mass loss, (0.22% and 0.49%). Subsequently, after drying in an oven at 100 °C, a small further mass loss was observed. In GRE-WI coating, being water based, lost ~4.2% mass. Visual inspection of the samples after water soak test showed not much change in GRE-EI and GRE-EDI surfaces and even in GRE-WI there was some coating there, the images are shown in the following section.

#### 2.3.3. Durability to Impact and Effect of Water Absorption on Impact Performance

**Figure 3:**
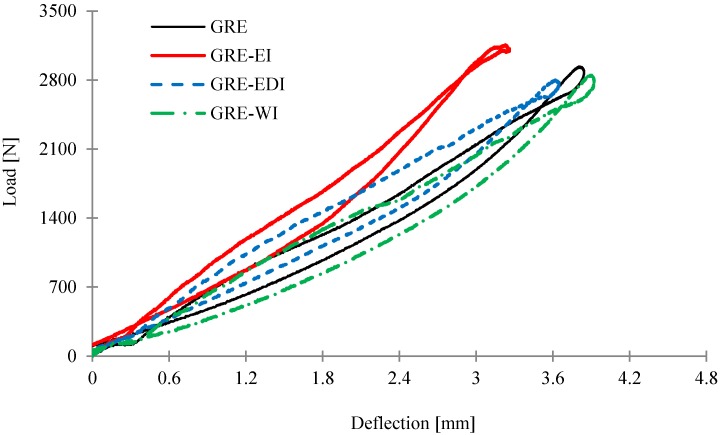
Load *versus* deflection curves of the control and intumescent coated samples from 5 J impact energy loading.

**Figure 4:**
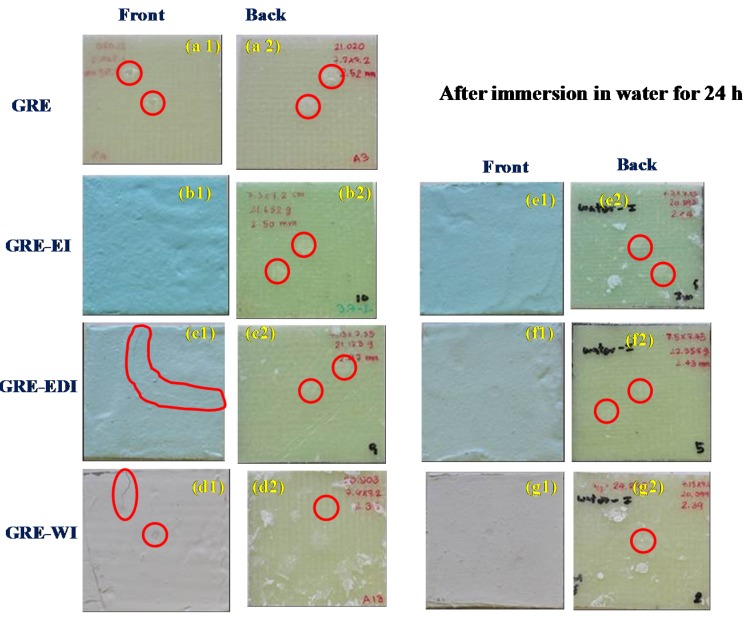
Images of impact damage on the front (impacted side)) (**a1**–**g1**), and back surfaces (**a2**–**g2**) of all the glass fibre-reinforced epoxy (GRE) samples after 5 J drop-weight impact tests. (**a**) GRE, (b) GRE-EI, (**c**) GRE-EDI, (**d**) GRE-WI, (**e**) GRE-EI-WS, (**f**) GRE-EDI-WS and (**g**) GRE-WI-WS. WS: water soaked.

The typical load-deflection curves for a control and coated sample obtained under 5 J impact energies are shown in Figure 3. The samples exhibit both the loading and unloading portions of the curve due to the effect of clamping and vibration [18]. The loading, shown as the upper portion of the curve, implies when the impactor hits the specimen, while the lower portion of the curve represents the behavior of specimen once the impactor rebounds away from the specimen during the unloading process. The results indicate that the coatings have no effect on impact properties of the laminate. This can be seen from the morphologies of post-impact samples examined using a digital camera and given in Figure 4 where it can be seen apart from small cracks in some coatings, the coatings are still intact and not debonded from the surface. The damage on the front surface seen on control GRE sample, cannot be seen in coated samples, however the damage on reverse side in all coated samples is similar to that observed in case of control samples.

The impact modulus (*E*_i_) of samples calculated from the initial load- deflection curves are given in Table 5, which shows a minimal effect of the coatings. It must be noted that for calculating the modulus, the thickness of the laminate was taken into account (see Section 3.6.3).

**Table 5:** The impact moduli of all coated samples before and after water soak test.

Sample	Coating Thickness (mm)	Impact Modulus (GPa)	Impact Modulus of Water Soaked Samples (GPa)
GRE	-	19.3 ± 0.5	-
GRE-EI	1.13 ± 0.12	20.6 ± 0.1	19.8 ± 0.6
GRE-EDI	1.15 ± 0.14	20.8 ± 1.3	18.6 ± 0.1
GRE-WI	0.78 ± 0.02	19.8 ± 1.2	17.6 ± 1.9

The water soaked samples from Section 2.3.2 were also subjected to impact tests. While there was minimal effect of impact on the EI coating, the impact modulus decreased in samples with EDI and WI coatings. This is consistent with water absorption results of these coatings as seen from Table 4, indicating that while there is no change in visual damage, the coatings become a bit softer, affecting the modulus.

### 2.4. Effect of Environmental Conditions on Resistance to Fire Performance of Intumescent Coatings

**Figure 5:**
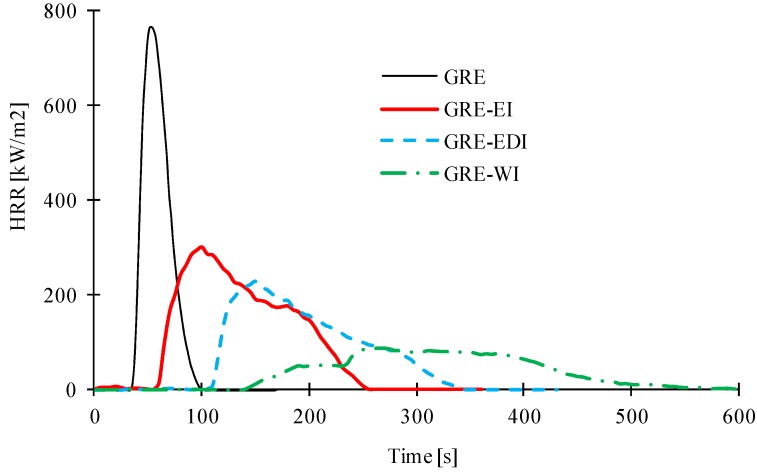
Heat release rate *versus* time curves of the GRE and coated samples at 50 kW*/*m^2^ heat fluxes with an ignition source.

The effects of water absorption and impact on the resistance to fire properties of the intumescent coating were also observed, for which all tested samples from above section were exposed to a cone calorimeter at 50 kW/m^2^. The heat release rate (HRR) *versus* time curves for control and intumescent coated samples are shown in Figure 5 and all derived results are given in Table 6. The control sample without any surface coating ignited after 36 s of continuous radiant heat exposure, after which period heat releases started rising and reached a peak heat release rate (PHRR) of 840 kW/m^2^ at 59 s, followed by a rapid reduction in the HRR, which signifies the cessation of the flaming combustion process. At the end of the experiment, all resin was burnt and no residual char was left to hold the glass fabric layers together. In all intumescent coated samples the time-to-ignition (TTI) was delayed, PHRR reduced and time-to-PHRR (TPHRR) delayed and also total heat release (THR) increased. This is typical behavior of samples showing passive fire protection [19,20]. With heat the intumescent reactions are activated and char is formed, which suppresses the burning of underlying laminate, resulting in a slow increase in HRR. As can be seen from Table 6, for TTI and TPHRR in the three coated samples the order is: GRE-WI > GRE-EDI > GRE-EI, whereas the PHRR is in the order GRE-WI < GRE-EDI < GRE-EI. These can be related to the char expansion of these coatings. The higher expanded char provides good thermal insulating effect.

In our previous work, published elsewhere [2] we have discussed the cone calorimetric results of 1, 3 and 5 mm thick coatings on GRE surfaces, where it was seen that all coated samples ignited and the thickness of the coating did not affect the TTI, however the PHRR was much lower than that of the control. The ignition was due to the resin binder present in the coating. In all coated samples two PHRR were seen, first one was very small and the second PHRR was delayed. With increasing thickness the first PHRR was not much affected, but the second PHRR was delayed and reduced in intensity. The results presented here for 1 mm thick coatings while have similar trends, are slightly different. In the present work there is only one peak and TPHRR is also reduced for each coating. This could be partly due to different flammability of the substrate, the GRE control sample and partly due to different batches of the coatings and coating thicknesses (variation due to hand coating). The cone calorimetric results of the GRE substrate in this case are 36 s TTI, 840 kW/m^2^ PHRR, 59 s TPHRR and 33.6 MJ/m^2^ THR compared to 49 s TTI, 733 kW/m^2^ PHRR, 91 s TPHRR and 38.8 MJ/m^2^ THR in our previous work [2]. This is due to different resin contents, *i.e.*, 60 wt % in this work and 50 wt % in the previous work. It is well known that flammability of the substrate affects the performance of the coatings.

**Table 6:** Cone calorimetric data for the control and all coated samples at 50 kW/m^2^ heat fluxes with an ignition source.

Samples	TTI (s)	FO (s)	PHRR (kW/m^2^)	TPHRR (s)	THR (MJ/m^2^)
GRE	36	105	840	59	33.6
GRE-EI	60	293	320	82	37.6
GRE-EI-WS	64	288	314	98	37.7
GRE-EI-ID	65	325	344	115	50.0
GRE-EI-WS-ID	45	350	305	115	50.1
GRE-EDI	96	369	239	130	35.8
GRE-EDI-WS	68	343	249	110	36.3
GRE-EDI-ID	29	395	225	115	50.1
GRE-EDI-WS-ID	65	330	333	110	46.4
GRE-WI	167	573	95	223	24.1
GRE-WI-WS	57	145	618	84	27.0
GRE-WI-ID	160	700	117	235	37.5
GRE-WI-WS-ID	60	175	716	95	38.3

Note: The cone results presented are reproducible to within ±10%. TTI: time-to-ignition, FO: flameout, PHRR: peak heat release rate, TPHRR: time-to-PHRR, THR: total heat release, WS: water soaked, ID: impact damaged.

#### 2.4.1. Effect of Water Absorption on Fire Performance

**Figure 6:**
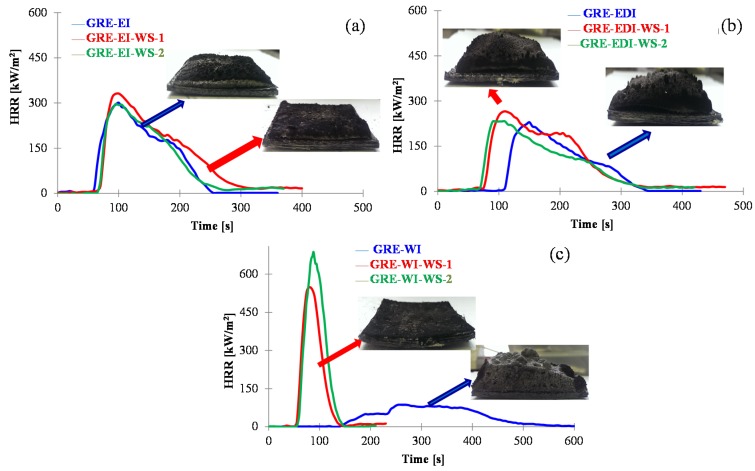
The heat release rate *vs.* time curves and images of residual char at 50 kW/m^2^ of the intumescent coated samples before and after water-soak (labelled as WS after the sample name) test: (**a**) GRE-EI, GRE-EI-WS-1/2, (**b**) GRE-EDI, GRE-EDI-WS-1/2 and (**c**) GRE-WI, GRE-WI-WS-1/2. WS: water soaked, 1 and 2 represent replicate tests 1 or 2.

The water soaked samples discussed in Table 4 were subjected to cone calorimetric tests. Their HRR *vs.* time curves are shown in Figure 6. For each sample two replicates of the water soaked tests are shown for reproducibility study. For epoxy based intumescent coated sample, EI, there is no significant effect on the TTI, PHRR and TPHRR after immersion in water when compared to the respective control sample. There is also not much difference in char expansion as shown by the inset images of control and water soaked samples. This relates to minimal effect of water absorption of this coating on mass loss of the coating and thickness change as seen from Table 4. The water absorption has more effect on the EDI sample (0.49% mass loss and 1.14% change in coating thickness on air drying) than in EI (0.22% mass loss and 0.52% change in coating thickness), which affects the fire performance. The TTI is reduced from 96 s in GRE-EDI to 68 s in GRE-EDI-WS, PHRR slightly increased (239 to 249 kW/m^2^) and TPHRR reduced from 130 to 110 s. It must be pointed out that this is not due to the DOPO chemical, which chemically reacts with the epoxy resin binder in the coating, but due to other components of the coating, which due to commercial sensitivity of the coatings are not discussed here. There is also reduction in char thickness of the coating as seen from Figure 6b. The water based coating WI, as expected lost all of its intumescing property, the char in water soaked samples (GRE-WI-WS) has no expansion and the TTI, PHRR *etc.* of these samples are similar to that of the control GRE sample discussed in Table 3.

#### 2.4.2. Effect of Impact on Fire Performance

**Figure 7:**
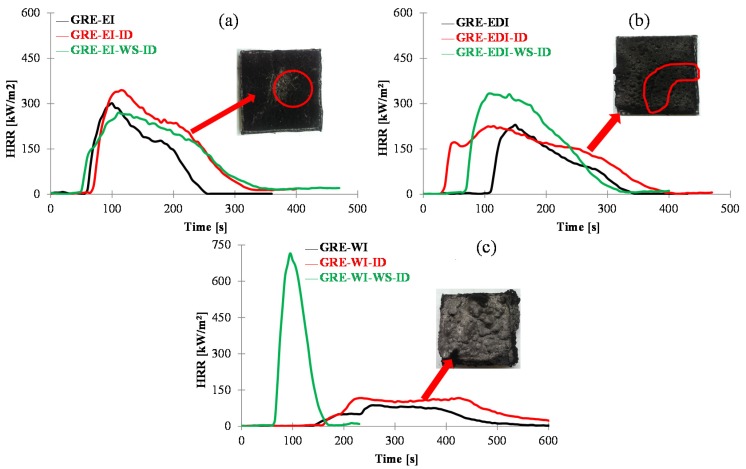
The heat release rate *vs.* time curves and images of residual char at 50 kW/m^2^ of the intumescent coated samples before and after impact ((labelled as ID after the sample name) and water soak plus impact tests (labelled as WS-ID after the sample name). (**a**) GRE-EI, GRE-EI-ID, GRE-EI-WS-ID, (**b**) GRE-EDI, GRE-EDI-ID, GRE-EDI-WS-ID and (**c**) GRE-WI, GRE-WI-ID, GRE-WI-WS-ID.

The effect of damage due to impact on the fire performance of the coatings can be seen from Figure 7. As discussed in Section 2.3.3, in epoxy based GRE-EI, there was not no damage to the coating (see Figure 4(b1)), which is reflected by minimal change in fire performance. There is no significant effect on TTI. PHRR is increased slightly, but the effect is minimal, considering ±10% reproducibility in cone calorimetric results. However, due to prolonged heating, THR is increased. The damage observed in the composite back surface in Figure 4(b2) can also be seen in the charred residue in Figure 7a. In GRE-EDI sample in the specimen where the coating was damaged (Figure 4(c1)), the effect on the fire performance is quite clear, *i.e.*, the TTI is decreased, though there is not much effect on the PHRR, but the THR is increased. This must be noted that this cracking pattern is not reproducible. This image is shown here to show that when the coating cracks, the fire performance is affected. In case of GRE-WI coating the small crack observed on the surface (Figure 4(d1)) is not big enough to affect the intumescence of the char (see Figure 7c) and hence the fire performance. In conclusion, the impact of ~5 J has a minimal effect on the fire performance of these coatings.

The effect of impact on water soaked test can be seen from the HRR *vs.* time curves in Figure 7 and results in Table 3, which is similar to that observed for water soaked samples, *i.e.*, in GRE-EI sample there is a minimal effect, in GRE-EDI, the TTI is decreased, PHRR increased and THR increased. In GRE-WI however, the sample behaves as an uncoated control sample. This means that the loss in intumescing and resistance to fire properties in water soaked and impacted sample GRE-WI (GRE-WI-WS-ID) sample is due to water and not impact.

### 2.5. Effect of Heat/Fire on Flexural Performance of Intumescent Coated GRE Composites

The fiber-reinforced polymeric composites used for structural applications are required to retain their mechanical integrity during and after exposure to heat/fire for a period of time to avoid catastrophic structural failure. This is important if they are to compete with metals. Metals can hold integrity for a long time whereas when resin parts (responsible for holding the fibers) of the composite softens, degrades, and burns, the fibers cannot hold the structure even for a short period of time. The effect of radiant heat on the mechanical properties of the GRE composites was investigated by exposing samples to a cone calorimeter at 50 kW/m^2^ heat fluxes for prescribed time periods (*i.e.*, 60 and 120 s), and then measuring the residual flexural modulus of the samples by a three point-bending test. These relatively short periods of thermal exposure were chosen as the laminate specimens being investigated in this work are thermally and physically thin. Prolonged thermal exposure to simulate practical fire scenarios in fire-prone engineering structures would have completely depleted all the flexural properties. However, since the post-fire flexural performance is considered on a comparative basis, the geometric aspect and thermal exposure periods are irrelevant. The flexural load was applied on the heat-damaged surface so that the damaged surface would bear the compressive strain. While calculating the residual flexural modulus, the original thickness of the sample was used.

The post-heat flexural moduli of all samples with/without surface coatings under varied thermal damage conditions are given in Table 7, which show that the control laminate when exposed to a 50 kW/m^2^ heat flux for 60 s, can retain only 61% of the modulus and by 120 s only 24% is retained. Severe delamination and loss in structural integrity of the composite laminate was observed. On the other hand, all intumescent coatings showed higher retentions in flexural modulus compared to the control sample, after 120 s GRE-EI, GRE-EDI and GRE-WI could retain 84%, 90% and 96% modulus, respectively. This behavior can be related to thermal barrier efficiency of these coatings discussed in Section 3.1, which is in the same order, *i.e.*, GRE-WI > GRE-EDI > GRE-EI.

**Table 7:** Post-heat flexural moduli of all the GRE samples with/without surface coatings under varied thermal damage conditions.

Sample	Exposure to 50 kW/m^2^					
	60 s			120 s		
	*E*_Un_ (GPa)	*E*_Dam_ (GPa)	Retention (%)	*E*_Un_ (GPa)	*E*_Dam_ (GPa)	Retention (%)
GRE	17.8	10.8	61	16.1	3.9	24
GRE-EI	17.7	16.5	93	16.3	13.8	84
GRE-EDI	17.1	16.8	97	16.1	14.6	90
GRE-WI	17.0	16.9	99	15.9	15.3	96

*E*_Un_ indicates the flexural modulus of the composite undamaged specimen; *E*_Dam_ indicates the flexural modulus of the composite damaged specimen; Retention in Flexural modulus (%) = $\frac{E_{\text{Dam}}}{E_{\text{Un}}}$ × 100.

## 3. Experimental Section

### 3.1. Materials

#### 3.1.1. Glass Fiber-Reinforced Epoxy (GRE) Composite

Epoxy resin system: epoxy phenol novolac resin (Araldite LY5052, Huntsman, Basel, Switzerland) and cycloaliphatic polyamine-2,2-dimethyl-4,4-methylene bis cyclohexylamine hardener (Aradur HY 5052, Huntsman, Basel, Switzerland). Glass fiber: woven rovig glass fiber of E-glass type (300 g/m^2^, Glasplies, Southport, UK).

#### 3.1.2. Intumescent Coatings

Three types of commercial intumescent coatings, two of which were epoxy resin based and one water based, supplied by Sherwin-Williams (formerly Leigh Paints), UK were used. Being commercial products, their intumescent components are not known. The details of the binder used in each type of coating are as:

(i) Epoxy based intumescent coating (EI): formulation comprises two component material:
  - Base resin containing an epoxy resin, ethyl hexyl glycidyl ether and bisphenol F-epichlorohydrin.
  - Hardener containing 2,4,6-tris(dimethylaminomethyl)phenol and triethylenetetramine.
(ii) Flame retarded epoxy based intumescent coatings (EDI): formulation comprises of two component materials.
  - Base resin containing an epoxy base resin, 9,10-Dihydro-9-oxa-10-phosphaphenanthrene-10-oxide (DOPO) modified epoxy resin complex, 1,4-bis(2,3epoxypropoxy) butane, and triphenyl phosphate.
  - Hardener containing, zinc borate, tetraethylpentamine and 3-aminopropyltriethoxysilane. The intumescent chemicals are same as in EI coating.
(iii) Water based intumescent coating (WI): This is a single component material, containing vinyl acetate/vinyl ester copolymer system, thermally active pigments, water, and butyl diglycol acetate.

### 3.2. Sample Preparation

#### 3.2.1. Glass Fiber-Reinforced Epoxy (GRE) Composite

Eight pieces of 300 mm × 300 mm woven E-glass fabric were used for composite preparation. The GRE composite samples of ~3 mm thickness were fabricated using a hand lay-up method by impregnating each glass fabric layer with the resin, using 30 wt % hardener with respect to resin, vacuum bagging and curing at room temperature (RT) for 24 h, and then post-curing at 80 °C for 6 h. The composition of the composite was: 60 wt % resin and 40 wt % glass fiber.

#### 3.2.2. Intumescent Surface Coating of Glass Fiber-Reinforced Epoxy (GRE) Composite

The GRE composites from Section 3.2.1 were cut into 75 mm × 75 mm size and then individually coated with three intumescent coatings to obtain 1, 3 and 5 mm coating thicknesses. The surface of GRE laminate samples was firstly cleaned with acetone, wiped very gently with a tissue and dried at room temperature for 10 min before any surface coatings. The coating process involved firstly preparation of different intumescent coatings formulations, EI, EDI and WI according to the manufacturer’s instructions and then each intumescent coating was independently applied on the GRE composite laminates’ surfaces by using paint brush and roller to get the uniform thickness. Since each sample was individually coated, the exact thicknesses of different coatings for different samples varied, hence the values are given for each test in relevant sections and tables. After coating the laminate’s surface, the coated laminates were cured at room temperature for 24 h, and then post-cured at 80 °C for 4 h in an oven.

#### 3.2.3. Intumescent Coating Samples for Char Expansion and Thermal Conductivity Measurements

For char expansion study, intumescent coating samples of circular shape with 25 mm diameter and of 1 mm thickness were used, whereas for thermal conductivity measurements samples were of 50 mm diameter round shapes with 5 mm thicknesses. Samples were prepared by pouring each formulation in aluminum molds of required dimensions and curing the coating similar to the procedure discussed in Section 3.2.2.

### 3.3. Thermal barrier and resistance to fire performance evaluation

The intumescent coated GRE composite samples were exposed to a cone calorimeter (Fire Testing Technology, East Grinstead, UK) at 50 kW/m^2^ heat flux in the horizontal mode at a distance of 25 mm from the cone heater with an ignition source according to BS 476-15:1993 [21]. The thermal barrier properties of all GRE samples were studied by using K-type thermocouples inserted in each sample, one on top of the surface coating, one underneath the coating and another one on the back surface of samples. The thermocouples recorded temperature as a function of time to get the temperature profiles of each coated sample. Two specimens were tested and the average temperature–time profiles are reported in this study.

Resistance to fire properties of the coatings was evaluated by the standard cone test according to BS 476-15. The different flammability parameters are reported as time-to-ignition (TTI), heat release rate (HRR), peak heat release rate (PHRR), time-to-PHRR (TPHRR) and total heat release (THR).

### 3.4. Char Expansion Study of Intumescent Coatings

#### 3.4.1. Char Expansion as a Function of Time Measurement using Infrared Camera in a Mass Loss Calorimeter

The expansion of three intumescent coatings as a function of time in the cone calorimetric equivalent test was conducted using a mass loss cone calorimeter (FTT) and a FLIR (ThermaCAM^™^ A40, FLIR Systems, Inc., Wilsonville, OR, USA) infrared camera, discussed in details elsewhere [2]. The test conditions used were similar to the ones used in cone experiment discussed above, *i.e.*, at 50 kW/m^2^ in presence of an electric spark igniter. The infrared camera was programmed to record temperatures between 50 and 1500 °C and images were recorded at 5 s intervals for test periods of 300 s for GRE composite samples and 1000 s for intumescent coatings. Two specimens of each sample were tested for reproducibility. The char expansion data were evaluated by using image analysis software (ThermoCAM Researcher program) from the movie obtained from the infrared camera and assuming one dimensional expansion.

#### 3.4.2. Char Expansion as a Function of Temperature Measurement using the Advanced Rheometric Expansion System (ARES)

An advanced rheometric expansion system (ARES 20A, Rheometric Scientific, TA Instruments, New Castle, DE, USA) with a concentric cylinder sample holder was used to evaluate the char expansion of the intumescent coatings as a function of temperature. The coating samples of circular shape with 25 mm diameter and of 1 mm thickness were used. All tests were performed under dynamic temperature ramp from 25 to 500 °C in strain-controlled mode over 1.5–3.0 gram-force range with 5 and 10 °C/min heating rates, 1.0 rad/sec frequency range and 1% strain limits. Three replicate specimens of each coating were tested and the results were averaged. The details are given elsewhere [2].

### 3.5. Thermal Conductivity Measurement

Thermal conductivities of three intumescent coatings and their expanded chars at different temperatures ranging from 20 to 700 °C were measured using a hot disk thermal constant analyzer (Hot Disk transient plane source (TPS) 2500 S, Thermo-concept, Bordeaux, France), which is a transient plane source technique [5]. The sensor which is a warmth emitter and thermocouple is directly embedded in the sample to ensure a good contact between the sample and the sensor during the experiment, the details are given elsewhere [2,5]. One sample of each type was tested. The sample was heated to a particular temperature and held at that temperature for 3–5 min before the reading was taken. The furnace is purged with flowing nitrogen to prevent the oxidation of the sensor. A cooling system is also connected to the furnace for measuring the thermal conductivity of the material at a particular temperature. The conductivity measurements were made by applying a power of 0.05–0.19 W for 10–80 s, depending on the thermal conductivity of each sample at a particular temperature. The temperature was raised to the next required value and thermal conductivity readings taken again. These experiments were conducted at room temperature, 200, 300, 400, 500, 600 and 700 °C. The reported thermal conductivity values at all temperatures are an average of three measurements taken at a particular temperature.

### 3.6. Effect of Environmental Conditions on Durability of Coatings

#### 3.6.1. Adhesion with the Substrate: Tape Pull Test

The adhesion between intumescent coatings and substrate was assessed by a tape pull test, similar to the one specified in ISO 2409:2007 [22], which are often used to examine the adhesion of films or sheets to an adhesive [23]. Initially, a piece of Sellotape^®^ (Winsford, UK) was applied on the surface of the coated laminate and smoothed with fingers to ensure good contact. Holding the sample with one hand, the tape was then peeled back at 180° angle in one smooth movement with the other hand. The test was repeated three times on different locations on the same sample. All samples were weighed before and after the test.

#### 3.6.2. Durability to Water Absorption

To observe the effect of moisture and water at atmospheric conditions on coatings, all coated samples were tested by the water-soak test, according to ISO 2812-2:2007 standard [24]. In each case two replicate specimens of 35 mm × 35 mm sizes were tested. As recommended in the ISO 2812-2:2007 standard, the four edges of all samples were sealed with an epoxy resin (AralditeLY5052 and Aradur 5052 hardener, Huntsman, Basel, Switzerland), the epoxy resin is the same as that used for the resin matrix of the GRE composite. All samples were first dried at 100 °C in an oven for 2 h, weighed and then fully immersed in 100 mL of deionized water. For each sample, a separate container was used and the water containers were covered with an aluminum foil. After keeping the samples for 24 h at room temperature, the samples were removed, dried at room temperature for 24 h and then at 100 °C for 2 h. All samples were then weighed again and images were taken with a digital camera to investigate the damage to morphologies of the coated surfaces after the tests.

#### 3.6.3. Durability to Impact

To investigate the impact resistance of the coatings, the coated samples (75 mm × 75 mm size) were tested using an Instron-Dynatup 9250 HV drop weight impactor (Instron Testing Equipment, High Wycombe, UK) with a 16 mm diameter hemispherical tup. This impact drop weight test is based on ASTM D7136 [25]. The steel impactor was of mass 4.62 kg and dropped from 110 mm height to produce an impact velocity of 1.46 m/s and 5 J impact energy loading. This impact condition was selected based on the damage (surface cracking) observed on control GRE composite after exposure to this condition. During the test, the high-speed data acquisition system (Dynatup^®^ Impulse^™^ software data capture system (Instron Testing Equipment, High Wycombe, UK)) has the capability of storing the entire impact event and hence recorded acceleration/ deceleration as a function of time. Using this data via numerical integration, the load-time, load-deflection, and energy-time curves were produced. The digital images of the samples after the impact tests were studied to investigate the damage to the coatings. Two replicate specimens of each sample were tested and then the impact modulus (*E*_i_) of each sample was calculated using Equation (1).

(1) $$E_{\text{i}}=\frac{3D^{2}}{4\pi h^{3}}K$$

where *D* = diameter of hole of the sample holder; *h* = thickness; *K* = initial stiffness determined from the load *vs.* deflection curve. For coated samples, the thickness of the substrate was used.

### 3.7. Flexural Performance of Heat/Fire Damaged Samples

The effect of one-sided radiant heating on the residual flexural moduli, *E*_f_, of GRE composite samples with and without surface coatings was investigated. Test specimens (125 mm × 20 mm × ~3 mm) were exposed to one-sided radiant heat fluxes of 50 kW/m^2^ from a distance of 25 mm for 60 and 120 s. The exposure times were selected based on the cone results to ensure that the control sample should show some degree of loss in its structural integrity. The heat-damaged specimens were allowed to cool down naturally to ambient temperature before the residual char was scraped off. The flexural moduli of all test specimens were determined prior to coating and following fire exposure via a three-point bending test. For heat-damaged GRE composite samples, bending loads were applied on the heat-exposed surface of the test specimen using a 100 N load cell Instron 3369 tensometer in the displacement control mode (cross-head speed of 1 mm/min). The flexural moduli values were then calculated using the Engineers’ bending equation [26]:

(2) $$E_{\text{f}}=\frac{l^{3}}{4bh^{3}}K$$

where *l* is the test span, *h* the thickness, *b* the width of the specimen and *K* is the flexural stiffness (e.g., the slope of the load-displacement curve). Two independent measurements were performed on each composite sample with the average data presented.

## 4. Conclusions

The results have demonstrated that the thermal barrier effectiveness of the intumescent coatings depends upon the degree of expansion and the thermal conductivity of the expanded char. By quantification of these two parameters the coatings of required thicknesses can be designed which would enable a composite structure to survive at defined heat flux for a specified period of time. It was observed that ~0.2 W/mK is the minimum thermal conductivity value of ~3 mm thick char that should be able to protect a composite structure from heat to maintain structural integrity for twice the time period than that of the uncoated sample. These thicknesses of chars can be obtained by 1 mm thick coatings of EI and EDI on GRE and 0.5 mm thick WI coating. That means coatings of at least 2–3 mm thickness would provide longer time to retain the mechanical properties. However, on a 3 mm thick laminate it is impractical to have a coating of >1mm thickness, hence rest of the work was done on 1 mm thick coatings. These could retain ≥ 84% flexural modulus of the GRE for about 120 s, while the control sample on similar exposure lost most of its strength and delaminated. In order to study other practical problems, the physical and durability of the intumescent coatings were also investigated by the tape pull method and the water-soak test. The results have shown that the coating layer on the surface was uniform and there was a strong adhesion between coating and the substrate in each coated sample. The water had minimal effect on the epoxy based coatings, whereas the water based coating lost most of its intumescing and thermal barrier properties. The impact of 5 J had minimal effect on the durability and fire performance of the coatings.

## References

[1] Ohlemiller T.J., Shields J.R. (1999). The effect of surface coatings on fire growth over composite materials in a corner configuration. Fire Saf. J..

[2] Luangtriratana P., Kandola B.K., Duquesne S., Bourbigot S., Duquesne S., Bourbigot S. (2015). Quantification of thermal barrier efficiency of intumescent coatings on glass fibre-reinforced epoxy composites. Flame Retardancy and Protection of Materials: Recent Advances and Current Perspectives.

[3] Camino G., Lomakin S., Horrocks A.R., Price D. (2001). Intumescent materials. Fire Retardant Materials.

[4] Le Bras M., Camino G., Bourbigot S., Delobel R. (1998). Fire Retardancy of Polymers: The Use of Intumescence.

[5] Jimenez M., Duquesne S., Bourbigot S. (2006). Characterization of the performance of an intumescent fire protective coating. Surf. Coat. Technol..

[6] Hao J., Chow W.K. (2003). A brief review of intumescent fire retardant coatings. Archit. Sci. Rev..

[7] Underwriters Laboratories Inc. (1994). Rapid Rise Fire Tests of Protection Materials for Structural Steel.

[8] BSI Standards Publication (2010). Test Methods for Determining the Contribution to the Fire Resistance of Structural Members. Applied Reactive Protection Products to Steel Member.

[9] International Organization for Standardization (2014). Fire Resistance Tests—Elements of Building Construction—Part 10: Specific Requirements to Determine the Contribution of Applied Fire Protection Materials to Structural Steel Elements.

[10] Sorathia U., Gracik T., Ness J., Durkin A., Williams F., Hunstad M., Berry F. (2003). Evaluation of intumescent coatings for shipboard fire protection. J. Fire Sci..

[11] Gardelle B., Duquesne S., Vandereecken P., Bourbigot S. (2014). Resistance to fire of silicone-based coatings: Fire protection of steel against cellulosic fire. J. Fire Sci..

[12] Duquesne S., Magnet S., Jama C., Delobel R. (2004). Intumescent paints: Fire protective coatings for metallic substrates. Surf. Coat. Technol..

[13] Bartholmai M., Schartel B. (2007). Assessing the performance of intumescent coatings using bench-scaled cone calorimeter and finite difference simulations. Fire Mater..

[14] Han Z., Fina A., Malucelli G., Camino G. (2010). Testing fire protective properties of intumescent coatings by in-line temperature measurements on a cone calorimeter. Prog. Org. Coat..

[15] Kandare E., Kandola B.K., Myler P., Edwards G. (2010). Thermo-mechanical responses of fiber-reinforced epoxy composites exposed to high temperature environments. Part I: Experimental data acquisition. J. Compos. Mater..

[16] Staggs J.E.J. (2010). Thermal conductivity estimates of intumescent chars by direct numerical simulation. Fire Saf. J..

[17] Gardelle B., Duquesne S., Rerat V., Bourbigot S. (2013). Thermal degradation and fire performance of intumescent silicone-based coatings. Polym. Adv. Technol..

[18] Sevkat E., Liaw B., Delale F., Raju B.B. (2009). Drop-weight impact of plain-woven hybrid glass–graphite/toughened epoxy composites. Comp. Part A Appl. Sci. Manuf..

[19] Kandola B.K., Bhatti W., Kandare E. (2012). A comparative study on the efficacy of varied surface coatings in fire proofing glass/epoxy composites. Polym. Degrad. Stab..

[20] Kandare E., Chukwudolue C., Kandola B.K. (2010). The use of fire-retardant intumescent mats for fire and heat protection of glass fibre-reinforced polyester composites: Thermal barrier properties. Fire Mater..

[21] BSI Standards Publication (1993). Fire Tests on Building Materials and Structures—Part 15: Method for Measuring the Rate of Heat Release of Products.

[22] International Organization for Standardization (2007). Paints and Varnishes—Cross Cut Test.

[23] Zhang Y., Hazelton D.W., Knoll A.R., Duval J.M., Brownsey P., Repnoy S., Soloveichik S., Sundaram A., McClure R.B., Majkic G. (2012). Adhesion strength study of IBAD–MOCVD-based 2G HTS wire using a peel test. Phys. C Supercond..

[24] International Organization for Standardization (2007). Paints and Varnishes—Determination of Resistance to Liquids—Part 2: Water Immersion Method.

[25] American Society for Testing Materials (2012). Standard Test Method for Measuring the Damage Resistance of a Fiber-Reinforced Polymer Matrix Composite to a Drop-Weight Impact Event.

[26] Tsai S.W., Hahn H.T. (1980). Introduction to Composite Materials.

